# Controlled decomposition of SF_6_ by electrochemical reduction

**DOI:** 10.3762/bjoc.16.244

**Published:** 2020-12-01

**Authors:** Sébastien Bouvet, Bruce Pégot, Stéphane Sengmany, Erwan Le Gall, Eric Léonel, Anne-Marie Goncalves, Emmanuel Magnier

**Affiliations:** 1 Université Paris-Saclay, UVSQ, CNRS, UMR 8180, Institut Lavoisier de Versailles, 78035 Versailles Cedex, France; 2 Electrosynthèse, Catalyse et Chimie Organique, Université Paris-Est Créteil, CNRS, ICMPE, UMR 7182, 2 rue Henri Dunant, 94320 Thiais, France

**Keywords:** electroreduction, fluoride anion, redox potential, sulfur hexafluoride

## Abstract

The electroreduction of SF_6_ is shown at ambient temperature in acetonitrile using an array of platinum microelectrodes to improve the electrical detection. Its half reduction potential occurs at −2.17 V vs Fc^+^/Fc. The exact number of electrons for the full consumption of sulfur hexafluoride was determined and this gas further quantitatively transformed into environmentally benign fluoride anion and sulfur by electrochemical reduction.

## Introduction

Sulfur hexafluoride (SF_6_) is a fluorinated gas firstly identified in 1900 by Henry Moissan [1]. The strategy applied at industrial level to obtain SF_6_ uses sulfur in the presence of molecular fluorine. Sulfur hexafluoride possesses the particularity of being an inert gas both chemically and physiologically, it is non-flammable, has a high density and a high dielectric constant (2.5 times greater than that of air) [2–5]. These properties explain that this compound is widely used industrially as an electrical insulating gas in circuit breakers or in electrical substations [6–7]. On the other hand, SF_6_ is a greenhouse gas [8]. It has indeed a global warming potential (GWP) 22,000 times greater than CO_2_ [9]. From an industrial point of view, this requires efficient methods of recycling or destroying SF_6_. This last point implies, because of its great stability, the use of expensive methods requiring a large input of energy (high temperature, high pressure). Many SF_6_ decomposition strategies so far developed use photoreduction, plasma discharges or even photolysis processes [10–11]. Beyond the energetic high cost of such processes, they produce side products that are highly reactive, corrosive and toxic [12]. Recent and really impressive works were devoted to the decomposition of sulfur hexafluoride using stoichiometric or catalytic amounts of metals (Rh, Ni, Pt) [13–16]. Organic derivatives (phosphines or bipyridine) proved efficient tools for the selective degradation of SF_6_ [17–18]. Other elegant approaches have described the use of SF_6_ as precursor of reagent for fluorination or pentafluorosulfanylation. Very interestingly, the photochemical activation of this gas was described and allowed the in situ transformation of alcohols into alkyl fluorides [19–20]. The modern and green photoredox catalytic activation of SF_6_ was recently performed for the fluoro- and alkoxypentafluorosulfanylation of styrenes [21–22]. The same type of transformation was also described through the reductive activation of sulfur hexafluoride with TEMPO [23].

To the best of our knowledge, electrochemical reduction of SF_6_ has not yet been disclosed. The decomposition of sulfur hexafluoride by electrochemistry can nevertheless be a suitable answer and interesting alternative to the previous expensive options. In this article, we describe the electrochemical behavior of sulfur hexafluoride dissolved in various organic solvents. After combining an analytical approach of electrochemistry and ^19^F NMR spectroscopy, we have succeeded in the total consumption of SF_6_ in an electrochemical cell.

## Results and Discussion

The first step of this work began with the measurement of the solubility of sulfur hexafluoride in an organic solvent. This data was not available in the literature but was however crucial for the implementation of the electrochemical experiences. Two solvents (DMF and acetonitrile) were selected for their good dissolution abilities and their large electroactivity area. The solubility of SF_6_ was measured, at 20 °C, by ^19^F NMR with chlorodifluoromethoxybenzene as internal standard probe. The concentration value for DMF was quite low (0.17 g/L) whereas the one for acetonitrile (2.48 g/L) was convenient for further studies. Acetonitrile is a common nonaqueous solvent in electrochemistry. Having a dielectric constant relatively high (ε = 38), acetonitrile allows a good dissociation of several salts providing the conductivity of the medium. The concentration of SF_6_ in the following studies was then around 1.7 × 10^−2^ M.

We then turned our attention to the determination of the reduction potential of SF_6_. To allow its electroreduction feasibility in acetonitrile, an electrochemical analytical approach was required [24]. This crucial step was supported by sensors which are based on a micro-disc-array of platinum ultramicroelectrodes (20 µm diameter) acting as multi-probe channels.

Due to their small size, microelectrodes provide electrochemical studies of very low concentrations of electroactive substances contained in a small amount of solvent. As a consequence, a conductive solvent is not required and a low concentration of conducting salt is sufficient. In contrast to macroelectrodes, the current density on microelectrodes is very high. This allows a better current sensitivity in electrochemical measurements (cyclic voltametrics, polarization curve …) providing the study of rapid electron and coupled chemical reactions. In comparison to macroelectrodes, their small size leads to a large decrease of the capacitive current and avoids IR drop effects. The array of Pt microdiscs offers the advantage of summing the current intensity of each microelectrode, thus increasing the sensitivity of the resulting current [25].

Whereas a planar diffusion is observed onto classical electrode (size >0.1 cm^2^), a hemispherical diffusion is expected with a microdisc electrode due to the contribution of the current diffusion by edge effects. The mass transport regime is then drastically modified on microdisc electrodes and can be adjusted in accordance with the polarization time by monitoring the potential scan rate. By cyclic voltametry, unlike macroelectrodes, the polarization of microelectrodes with a low potential scan rate (≤25 mV/s) leads to a drastic decrease of Cottrel contribution which involves the disappearance of current waves. The resulting current becomes a stationary current of diffusion (*id*), which is directly proportional to the bulk concentration of the electroactive substance (*C*^∞^), its diffusion coefficient in the solvent (*D*), the number of electrons exchanged in the electrochemical process (*n*), and the radius of the microelecrode (*r*) and *F* the Faraday constant, in accordance with the following relation [26]:

[1] $$id=\;4nFD\;C^{\infty }r$$

**Figure 1:**
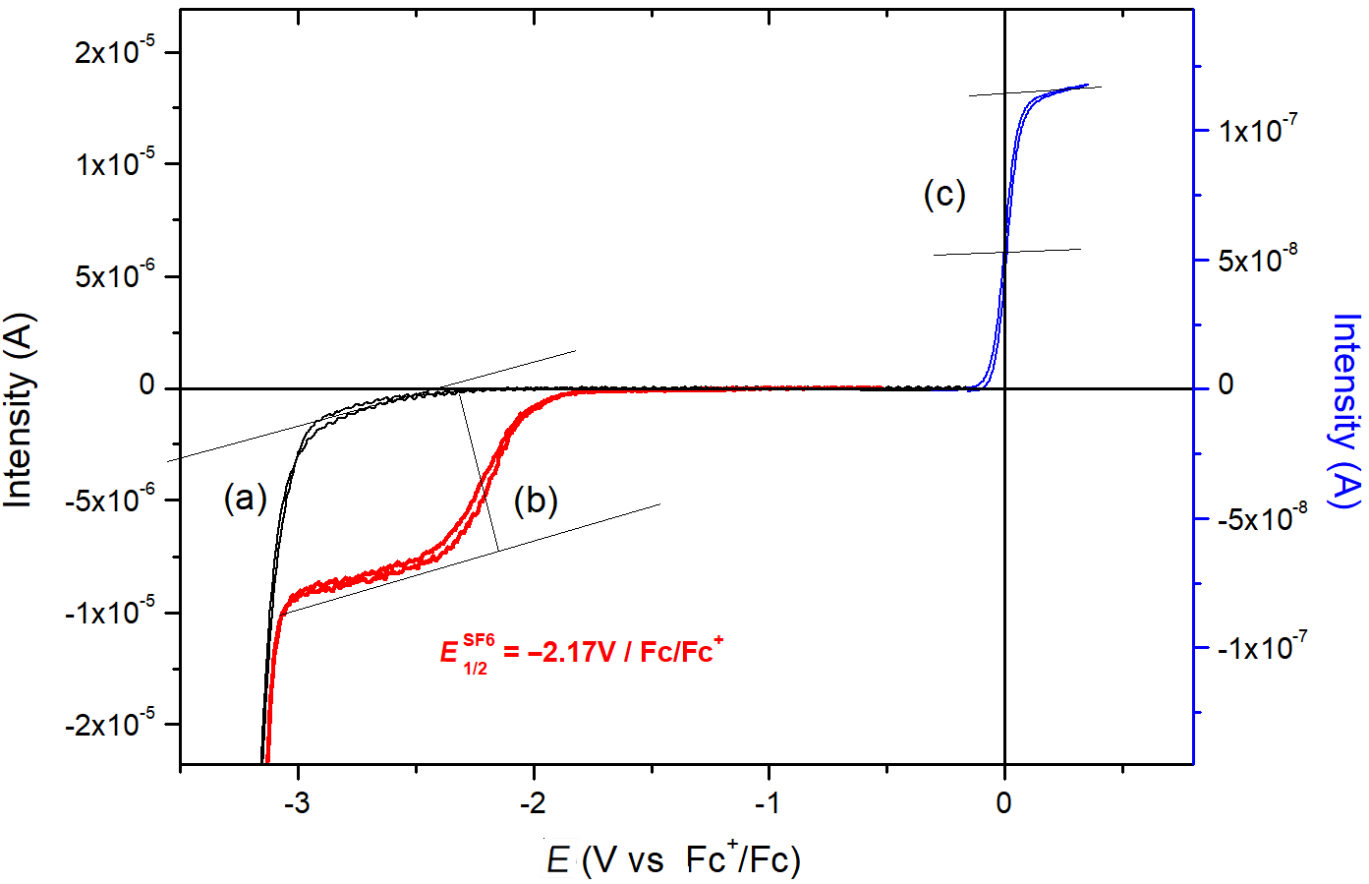
(a) Cyclic voltammetry onto microelectrode arrays (Ø = 20 µm) in acetonitrile freshly distilled after the addition of TBAClO_4_ (0.1 M) at room temperature, under slight argon stream, *v* = 20 mV/s. (b) Same conditions as for (a) after addition of SF_6_. (c) Same conditions than (a) after addition of ferrocene (10^−3^ M).

To the half limiting current (*id*/2) can be associated the half wave potential noted *E*_1/2_. For reversible electrons exchanged at the interface electrode/electrolyte, the half wave potential can be assimilated to the reversible formal potential redox couple noted *E*°ox/red [27]. After the addition of SF_6_, a stationary current is clearly observed before the acetonitrile reduction [28]. The electrochemial response of SF_6_ is related to a reproducible stationnary cathodic current. The intensity of the limiting current (plateau intensity) directly depends on SF_6_ concentration. From the voltamogram, the half wave potential of SF_6_ reduction is deduced and corresponds to −2.17 V vs Fc^+^/Fc (Figure 1).

To the best of our knowledge, this was the first determination of the redox potential of sulfur hexafluoride. Pleasingly, this value was compatible with the employed solvent and offered the opportunity to reduce this gas in solution. In order to reach our target, i.e., the total consumption of SF_6_ into stable and nontoxic species, the next step was the determination of the number of electrons. It was determined by using two controlled size (S) of Pt electrodes: Pt ultramicroelectrode (∅ 20 µm, S_1_ = 3.14 × 10^−6^ cm^2^) and Pt macroelectrode (∅ 0.76 mm, S_2_ = 4.5 × 10^−3^ cm^2^). Using Pt ultramicroelectrode, the stationary current density (*j*_1_) is deduced from Equation 2:

[2] $$j_{1}=\frac{4\times n_{e}{}_{-}FD[{\text{SF}}_{6}]}{\pi \times r}$$

Assuming a rapid electron transfer, the current (*j*_2_) decreases exponentially with time (*t*) according to the Cottrell law (Equation 3) onto a Pt macroelectrode, under sufficient electrochemical polarization [29].

[3] $$j_{2}=\frac{n_{e}{}_{-}FD^{\frac{1}{2}}S_{2}[{\text{SF}}_{6}](\pi t^{-\frac{1}{2}})}{S_{2}}$$

From a combination of Equation 2 and Equation 3, the determination of the number of electrons exchanged is given by Equation 4 [30]:

[4] $$n_{e}{}_{-}=\frac{4\times t\times j_{2}^{2}}{F\times [{\text{SF}}_{6}]\times r\times j_{1}}$$

**Figure 2:**
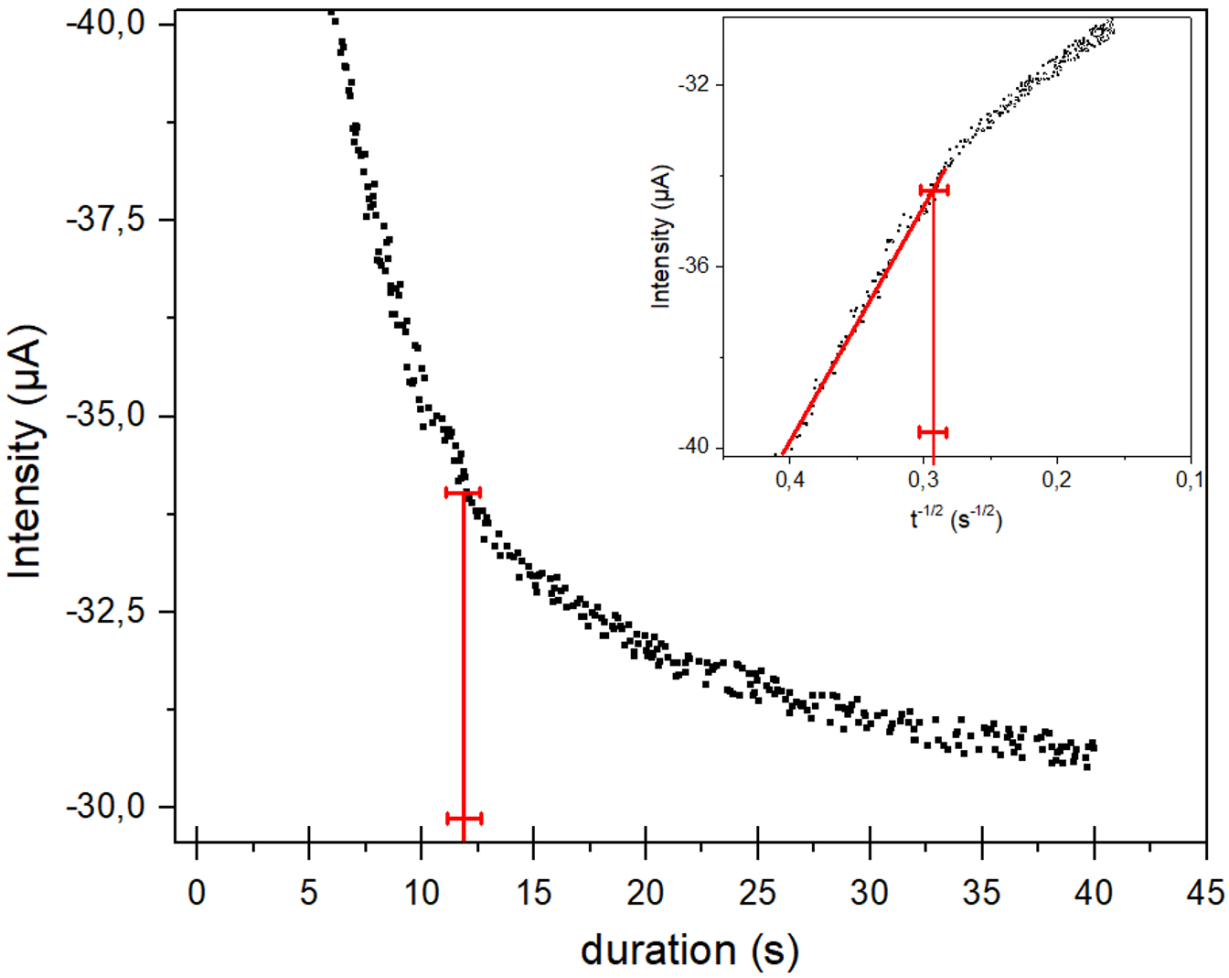
Variation of the current reduction (*i*_2_) of SF_6_ onto Pt macroelectrode (Ø = 0.76 mm) at −2.3 V vs Fc^+^/Fc in acetonitrile freshly distilled after addition of TBAClO_4_ (0.1 M) at room temperature, under slight argon stream. Insert figure: Determination of the upper limit on time of Cottrell law from the straight line *i_2_* = f(*t*^−1/2^).

The number of electrons exchanged (Equation 4) only depends on the validity of the Cottrell equation onto the macroelectrode (*j*_2_) since a constant current density (*j*_1_) is detected onto the microelectrode. The validity of the Cottrell equation requires a linear variation of the current density (*j*_2_) with *t*^−1/2^. This linear variation gives the upper limit on time. From this straight line the maximum duration (*t*) and the corresponding value of *j*_2_ are determined. These two values (*t* and *j*_2_) are then included in Equation 4 for the determination of the number of electrons exchanged. The macroelectrode (Ø = 0.76 mm) was polarized at −2.3 V vs Fc^+^/Fc after saturation of SF_6_ in the electrolyte. The chronoamperogram was reported in Figure 2. As it was expected, an exponential decrease of the current was indeed observed just before reaching a stationary current which was related to a constant layer thickness of SF_6_ diffusion. Indeed, the decrease in current comes from the consumption of SF_6_ at the interface electrode/acetonitrile. The current intensity depends on the SF_6_ flow in agreement with the 1st Fick’s Law from which the Cottrell equation is originated [29].

The upper limit on time was determined from the straight line reported in the insert figure (Figure 2). A value of 11.9 ± 0.7 s was deduced and the corresponding value of *i*_2_ (−34.2 µA ± 0.5) was determined from the chronoamperogram (Figure 2). Based on these values, the number of electron (*ne*^−^ = 7.8 ± 0.3) was deduced from Equation 4. The reduction mechanism of SF_6_ can then involve 8 electrons per molecule:


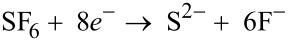


**Figure 3:**
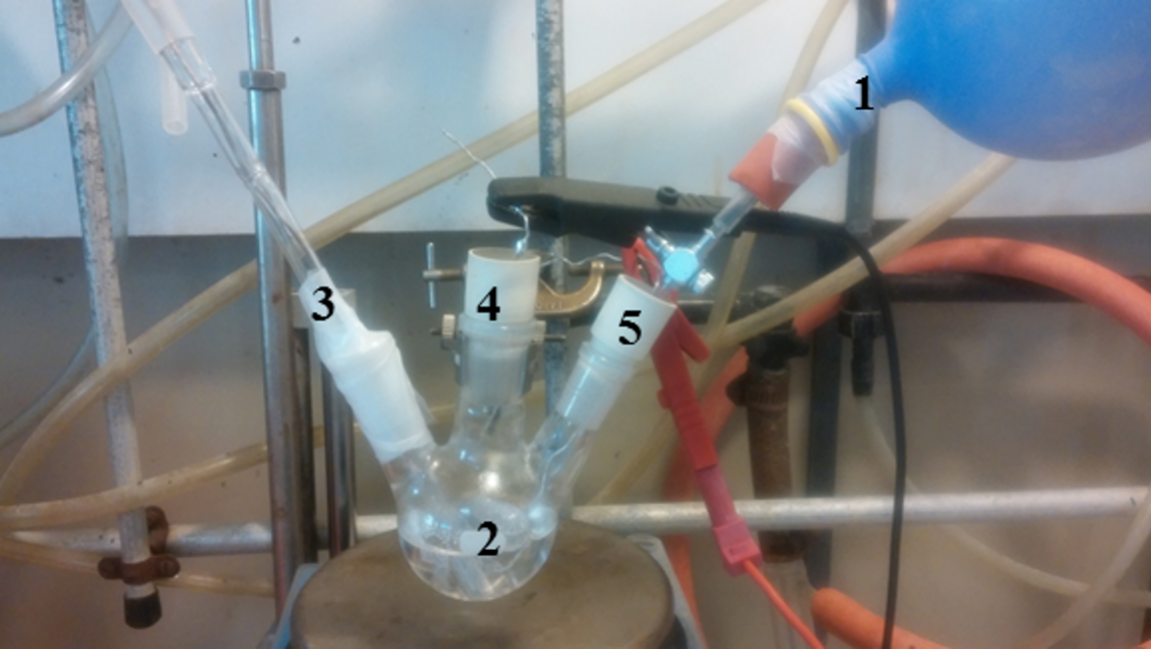
Single compartment three-electrode experiment. 1: Balloon of SF_6_, 2: electrochemical cell, 3: reference electrode (silver wire), 4: counter-electrode (platinum), 5: working electrode (platinum).

This preliminary study is an important step to determine the best conditions for the SF_6_ electrolysis (the choice of potential polarization, the understanding of current decrease). With these analytical data in hands, the large scale decomposition of SF_6_ was then undertaken. The reactions were carried out in a single compartment with a conventional three-electrode arrangement: two platinum electrodes and one silver reference electrode, SRE (Figure 3). The electrolysis is performed at constant potential (−2.3 V vs Fc^+^/Fc) with a continuous supply of SF_6_ placed in a rubber balloon (constant bubbling).

**Figure 4:**
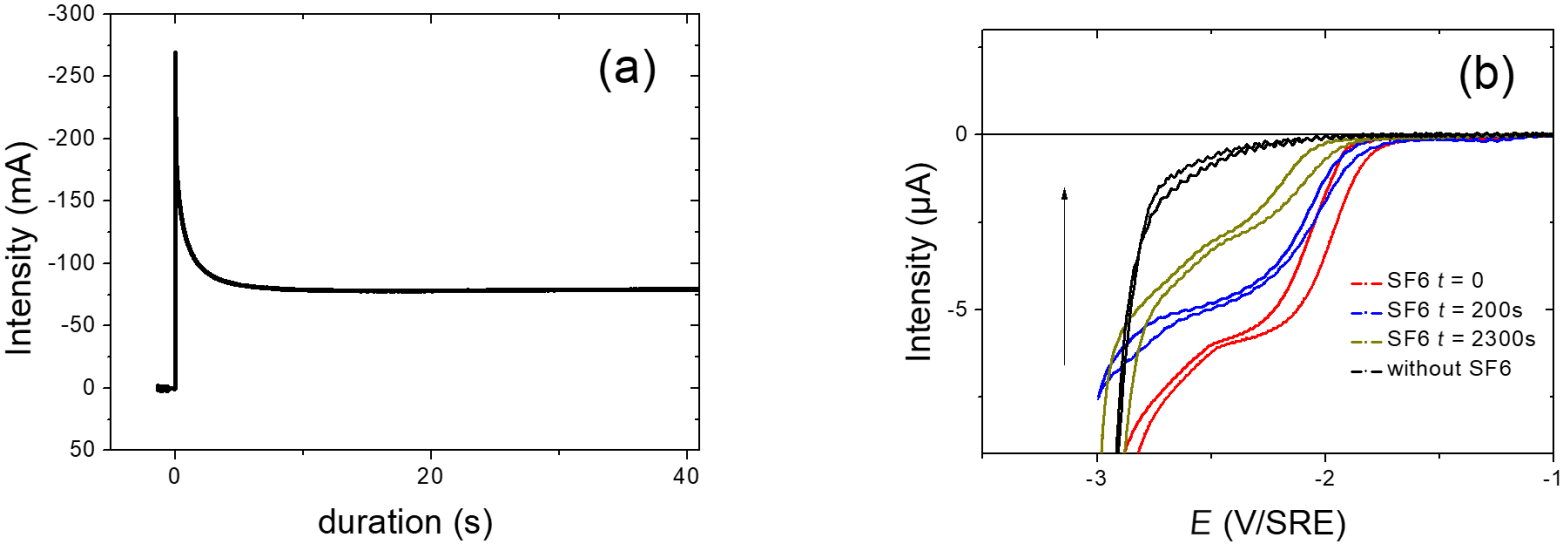
Electrolysis of SF_6_ at −2.3 V vs Fc^+^/Fc in acetonitrile freshly distilled after addition of TBAClO_4_ (0.1 M) at room temperature under slight argon stream. (a) Exponential decrease of the cathodic current according to the electrolysis duration onto a smooth Pt electrode (15 cm^2^). (b) Decrease of the cathodic current during the electrolysis duration which is detected onto an array of Pt microelectrodes (Ø = 20 µm).

The electrolysis was investigated on the same electrolyte but with larger surface of Pt electrode (15 cm^2^) (Figure 4). Figure 4b clearly highlights the consumption of the gas by the decrease of the electrochemical waves.

**Figure 5:**
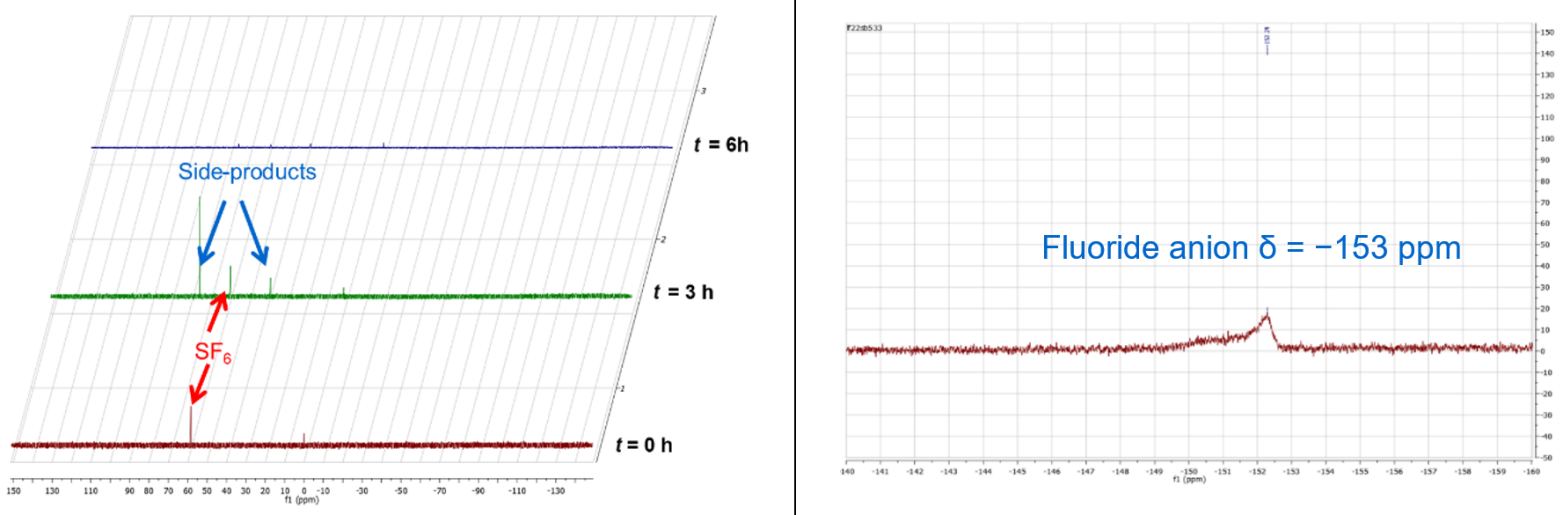
^19^F NMR evolution of the crude mixture along the time after electrolysis realized at constant potential of −2.3 V/SRE; (left). ^19^F NMR expansion of the final mixture.

The control of the decomposition of the sulfur hexafluoride was also monitored by ^19^F NMR (Figure 5). After 3 hours experience, unidentified side-products were detected by NMR. Identification of these fleeting species as well as their potential reactivity are under current investigation in our laboratory. The left part of Figure 5 clearly demonstrates the total disappearance of SF_6_ after 6 hours as well as all the fluorinated organic compounds. The only peak detected by ^19^F NMR is around −153 ppm. This value corresponds to the classical chemical shift range of a fluoride anion. Due to its broad appearance, we can postulate the association with cations coming from the supporting electrolytes based on tetrabutylammonium (TBA) structures after anion exchange. This poor resolved signal is quite classical for such species due to hydrogen bonds. Another important point is the production of H^+^ ions at the counter electrode because of the oxidation of acetonitrile [31–32]:


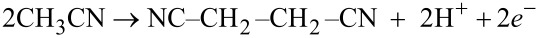


They can associate themselves with the F^−^ anions and generate bifluoride HF_2_ anions or even polyfluorides F(HF). The presence of fluoride anions can produce a Hoffman elimination on the alkyl chain of TBA giving rise to tributylamine, butene, and HF. We can suppose that the anion S_2_^−^ could also be react with these hydrogen sources and become H_2_S. Nevertheless, in spite of our efforts it is very difficult to clearly identify all these decomposition compounds. A comprehensive analytical study could be of interest but it falls down the scope of the present article.

## Conclusion

The smooth and controlled decomposition of sulfur hexafluoride was described under electroreduction. The reduction potential was firstly determined and used for preparative studies. The extrapolation on large scale of this methodology is under current development in our laboratory.

## Experimental

Acetonitrile (HPLC grade, Mallinckrodt) was distilled from CaH_2_ and then degassed using three freeze-pump-thaw cycles before use. An electrochemical cell filled up with 40 mL of acetonitrile with TBABr (0.1 M) or TBAClO_4_ (0.1 M) as conducting salt at room temperature. The degassing of the medium was performed under an argon stream before the bubbling of SF_6_ to reach a saturated concentration that is 1.7 × 10^−2^ M. A balloon filled with 8 g of SF_6_ (0.055 mmol) is connected to the device (see Figure 3) and the electrolysis is carried out until the balloon is empty. The electrochemical set-up was a classical three electrodes device. All potentials were measured against a pseudo silver reference electrode (SRE). The addition of ferrocene in the electrolyte gives access to an internal reference [33]. In order to improve the current detection level of SF_6_, platinum disk electrodes were used as working electrode (Pt ultramicroelectrode with a diameter of 20 µm, an array of eight Pt ultramicroelectrodes with a diameter of 20 µm and a Pt macroelectrode with a diameter of 1 mm). Platinum disk microelectrodes were made by sealing into very fine glass, one or eight platinum wires, with a diameter of 20 µm, into the same soft glass tubing [33]. The microelectrodes array was polished successively on finer grades sand paper. After the analytical approach, a Pt wire (15 cm^2^) is used to allow SF_6_ electrolysis.

Whatever the step of the study (analytical, electrolysis) a smooth platinum electrode with a larger surface was used as a counter electrode. Electrochemical measurements were covered by a wide range of a potentiostat for more effective current detection. A potentiostat–galvanostat such as Princeton Applied Research Model 263 was monitored by its front panel and analogue-to-digital conversion was provided by «Powerlab». A Parstat 2273 potentiostat was also used with its internal «Powersuite» software. For all cyclic voltammetries, the conventional representation of anodic currents is reported as positive values and cathodic currents as negative values.

SF_6_ electrolysis was performed in acetonitrile with a constant potential in an undivided electrochemical cell.

The control of the decomposition of sulfur hexafluoride was monitored by ^19^F NMR. The spectra were recorded with a Bruker AC-200 or AC-300 spectrometer. Reported chemical shifts are based on a first order analysis. Internal reference was the peak of CFCl_3_ (δ = 0.00 ppm) for ^19^F (188 or 282 MHz) NMR spectra. The ^19^F NMR of the final mixture was recorded after concentration under vacuum to see a broad fluoride signal.
